# Supplementary material for: Enabling nanoscale flexoelectricity at extreme temperature by tuning cation diffusion
Source: Nat Commun. 2018 Oct 25;9:4445. doi: 10.1038/s41467-018-06959-8 (PMC6202390; doi:10.1038/s41467-018-06959-8)
Supplement: Supplementary file 1 — Supplementary Information [file 41467_2018_6959_MOESM1_ESM.pdf]

Supplementary Information

Enabling nanoscale flexoelectricity at extreme temperature by tuning cation diffusion

Authors: L. Molina-Luna *et al*

Supplementary Note 1 Synthesis route for the formation of $0.75Na_{1/2}Bi_{1/2}TiO_3$–$0.25SrTiO_3$ (NBT-25ST) core-shell nanoparticles inside a transmission electron microscope

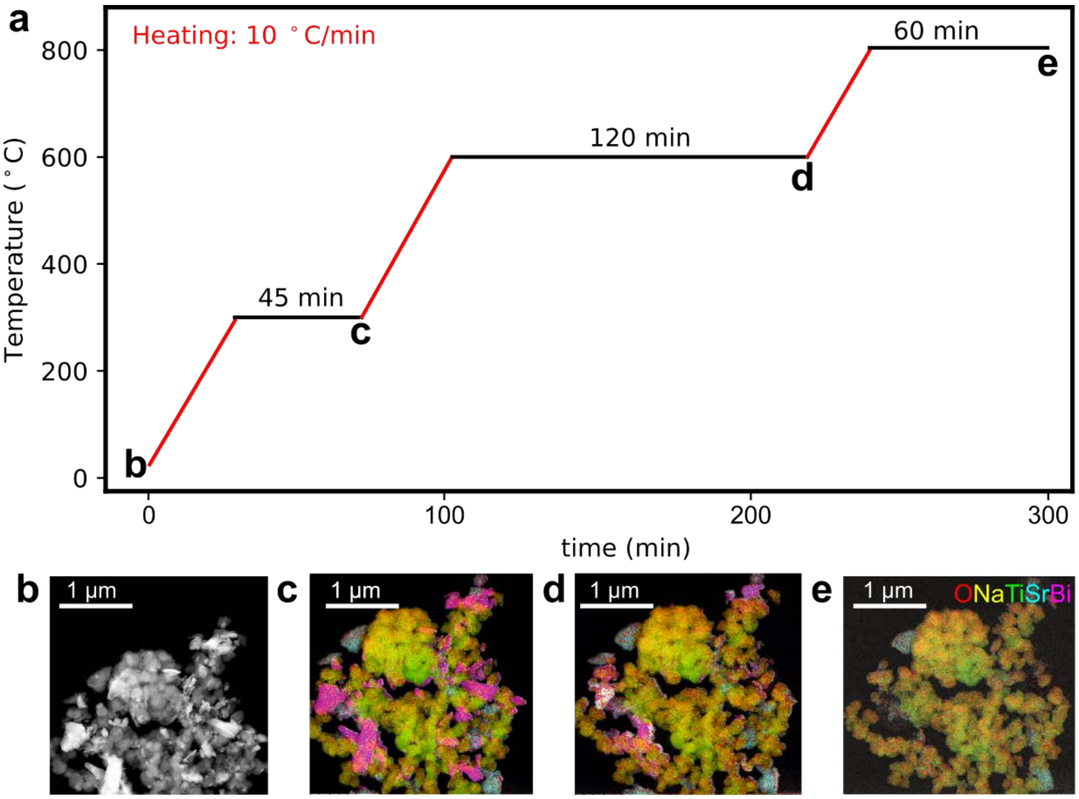

Supplementary Figure 1. In situ synthesis and characterization of the sample. a, Temperature-time program used to synthesize $0.75Na_{1/2}Bi_{1/2}TiO_3$–$0.25SrTiO_3$ core-shell nanoparticles via a solid-state chemical reaction inside the transmission electron microscope (TEM). **b,** High angle annular dark field (HAADF) Scanning TEM image of the mixed initial powders. **c-e,** Composite Scanning Transmission Electron Microscopy Energy-Dispersive X-ray Spectroscopy STEM-EDS maps obtained after each temperature step (red: O-K, yellow: Na-K, green: Ti-K, blue: Sr-K and purple: Bi-M, where K stands for and M stands for. The fabrication process is based on previous differential thermal analysis (DTA) and X-ray diffraction studies described in J. Koruza *et al*[1].

Supplementary Note 2 Electrostriction contribution to the polarization in core-shell NBT-25ST structure

The following note is based on the fruitful discussion with one of the reviewers concerning the electrostriction contribution to the polarization observed. We try to identify the value of electrostrictive-related polarization through the finite element calculation.

Here, we use the finite element simulation to calculate the distribution of the polarization with only electrostriction considered. The total energy is given in the following form:

$$H(\varepsilon_{ij}, E_i) = \frac{1}{2} C_{ijkl}(\varepsilon_{ij} - \varepsilon_{ij}^0)(\varepsilon_{kl} - \varepsilon_{kl}^0)$$

where C_{ijkl}, ε_{ij} and ε_{ij}^0 are elastic stiffness, total strain and eigenstrain respectively. The eigenstrain is quadratically related to the field,

$$\varepsilon_{ij}^0 = \frac{\kappa^2}{2} Q_{ijkl} E_k E_l$$

where κ is the material permittivity, Q_{ijkl} is the electrostrictive coefficient and E_i is the electric field. From the energy function, constitutive relations can be obtained as:

$$\sigma_{ij} = \frac{\partial H}{\partial \varepsilon_{ij}} = C_{ijkl}(\varepsilon_{ij} - \varepsilon_{ij}^0)$$

$$D_i = -\frac{\partial H}{\partial E_i} = \kappa E_i + \kappa^2 Q_{ijkl} E_j \sigma_{kl}$$

The electrostriction-related polarization is

$$P_i^0 = \kappa^2 Q_{ijkl} E_j \sigma_{kl}$$

We gave the following input values of Q_{11} = 0.02 m^4 C^{-2}, Q_{12} = -0.005 m^4 C^{-2} and Q_{44} = 0.01 m^4 C^{-2} for the core and doubled for the shell (these values are typically found for NBT-based material. See ref. Zhang *et al. Adv. Mater.* **21**(46), 2009). The permittivity of the core-shell nanoparticle was 1000 times the value of the vacuum. We set the electric field to 20 kV/mm by providing this voltage difference between the two boundaries.

Shown in Supplementary Figure 2 are the corresponding potential and electric field distributions. Supplementary Figure 3 shows the distribution of the electrostrictive-related strain. We can find that some relatively high strain region can only be found at the corner of the particle and the interface between the core and the shell. The magnitude of the strain here is 1% of the strain induced by the Sr^{2+} chemical gradient shown in the manuscript. Supplementary Figure 4 gives the electrostriction-induced polarization P_i^0. The color indicates the magnitude of the polarization and the arrow shows its direction. We found the magnitude of the polarization is below 0.0002 C m^{-2}, which is ~1.5% of the flexoelectric-based polarization (0.03 C m^{-2}). The large flexoelectric-based polarization is due to the size effect: as the scale down to nanometers, the strain gradient is much higher, thus the flexoelectric effect is more predominate.

We agree that electric field may affect distribution of the strain (and therefore pattern of nanoregions) through electrostrictive effect if nanoregions have anisotropic shape, but this effect is not comparable with flexoelectric effect.

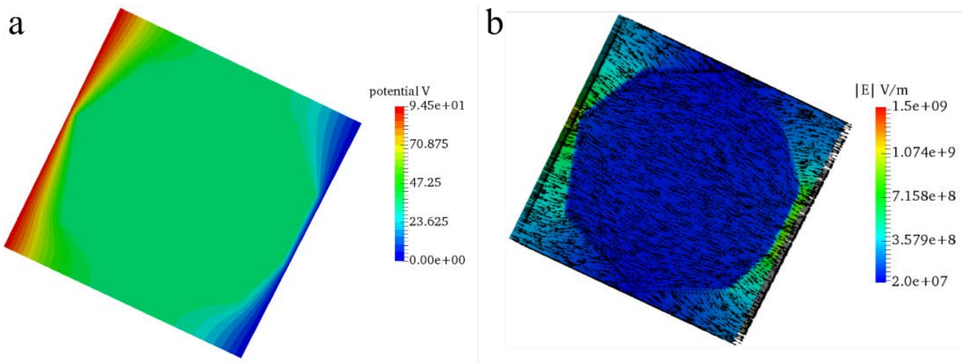

Supplementary Figure 2. Potential (a) and electric field (b) distribution of the nanoparticle.

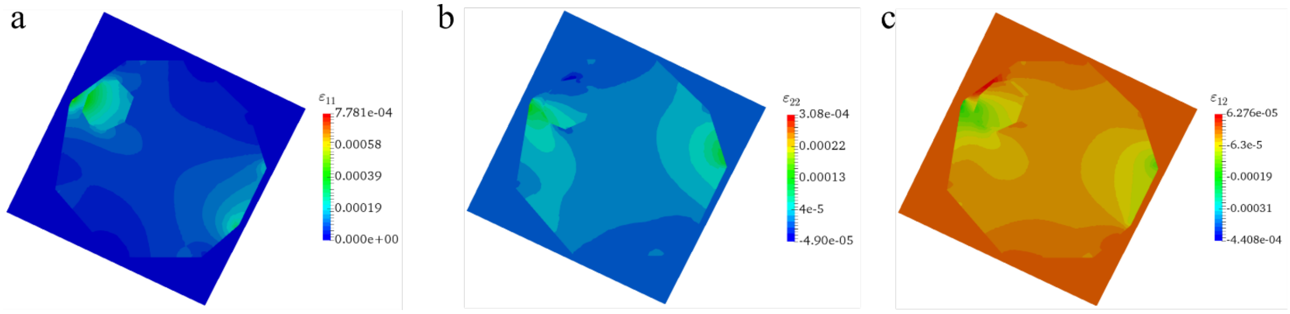

Supplementary Figure 3. Electrostrictive-related eigenstrain distribution. a to **c** show the strain component ε_{11}, ε_{22} and ε_{12}, respectively.

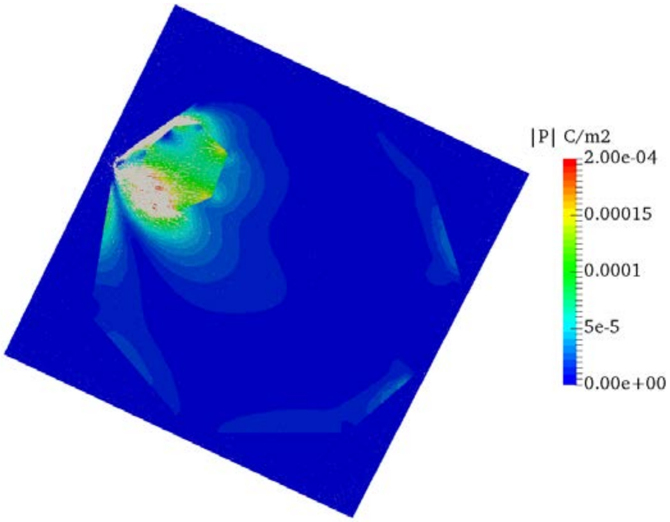

Supplementary Figure 4. Electrostriction-induced polarization distribution.

Supplementary Note 3 High-resolution TEM image and corresponding geometrical phase analysis (GPA) evaluation at the core-shell interface

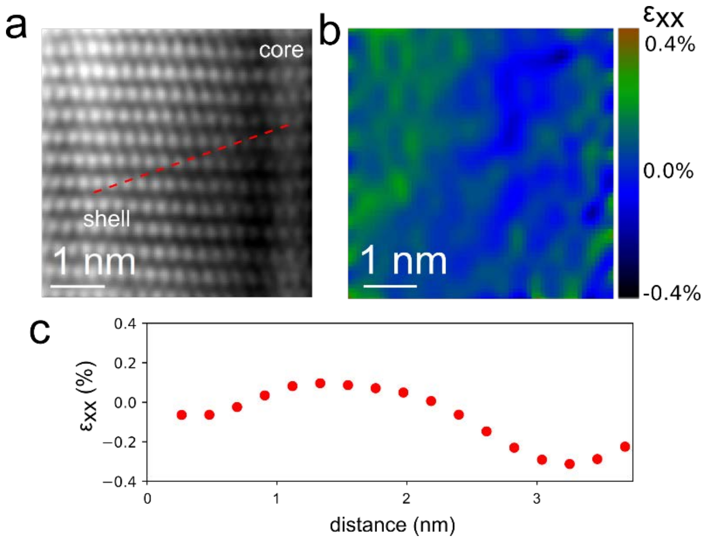

Supplementary Figure 5. Strain distribution at the core-shell interface. a, High-resolution transmission electron microscopy (TEM) image of the core-shell interface denoted in the manuscript Figure 4a. The image was Wiener filtered for noise reduction.[3] **b,** Corresponding geometrical phase analysis (GPA) evaluation map from high-resolution images[4] shown in **b**. **c,** The distribution of ε_{xx} along the red dashed line shown in **a**.

Supplementary Note 4 Switchable flexoelectric based polarization at extreme temperature with varying electric field

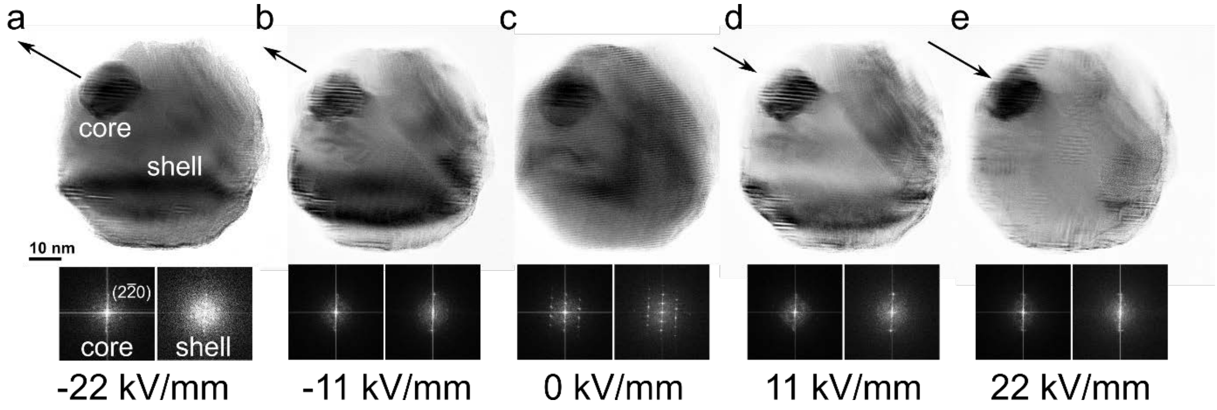

Supplementary Figure 6. TEM image and FFTs for core-shell nanoparticle under electric field. a-e The transmission electron microscopy (TEM) bright-field images of the core-shell NBT-25ST nanoparticle obtained at T = 800 °C. E = -21.9 kV mm^{-1} to +21.9 kV mm^{-1} and the corresponding Fast Fourier Transform (FFT) of core and shell. The arrow indicates the direction of the poling field.

Supplementary Note 5 STEM-EDS of a Bi-deficient 75 mol % $Bi_{0.49}Na_{1/2}TiO_3$-25 mol % $SrTiO_3$ nanoparticle

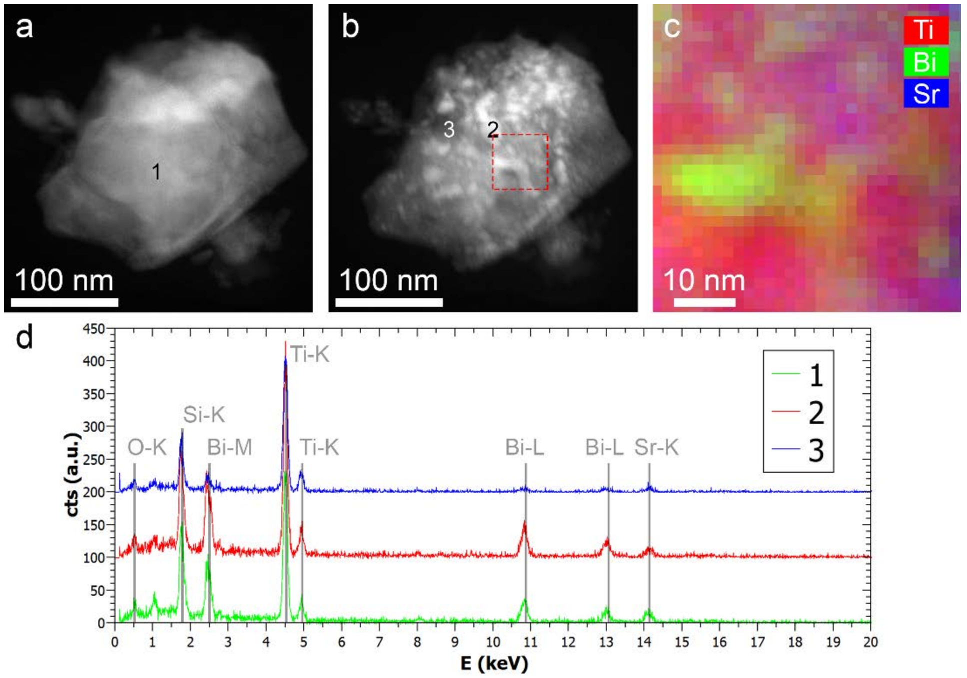

Supplementary Figure 7. Elemental mapping of the Bi-deficient nanoparticle. High angle annular dark field (HAADF) Scanning transmission electron microcopy (STEM) image of a Bi-deficient 75 mol % $Na_{1/2}Bi_{0.49}TiO_3$-25 mol % $SrTiO_3$ nanoparticle taken **a,** before heating **b,** after 10 min at 800 °C. **c,** RGB color map of the red box area (highlighted in **b**), RGB color coded to the elements Ti, Bi and Sr respectively. **d,** Energy-dispersive X-ray spectra at the marked points in a and b. The numbers 1 2 and 3 shown in **b** and **c** indicate the location where the spectra in **d** were recorded.

High angle annular dark field (HAADF) Scanning transmission electron microcopy (STEM) image of Bi-deficient NBT-ST nanoparticle is shown in Supplementary Figure 7a (before heating) and in Supplementary Figure 7b (after heating). Before heating, the powder is homogeneously mixed. After heating, the NBT-ST solid solution is formed. The energy dispersive spectrum (EDS) of the point in the center of the particle before heating (1), point in bright region after heating (2) and point in dark region after heating (3) shows no difference in element concentration, which means no core-shell structure is formed. The red box area shown in in Supplementary Figure 7b is 28 x 28 px² (50x50 nm²). The RGB color map was acquired (probe 4C, CA 50 µm, dwell time 3 s) after sintering, the color code as denoted in the legend. As shown in in Supplementary Figure 7c, we still resolve nanometer-scale segregated areas, but no defined chemical gradient is present. There is no defined large strain gradient generated by the Sr^{2+} diffusion. From our finite element simulation, there should be no domain-like nanoregions (DLNRs) induced by the flexoelectric effect.

Supplementary Note 6 Phase field simulation of the nanoparticle with different temperature and eigenstrain values

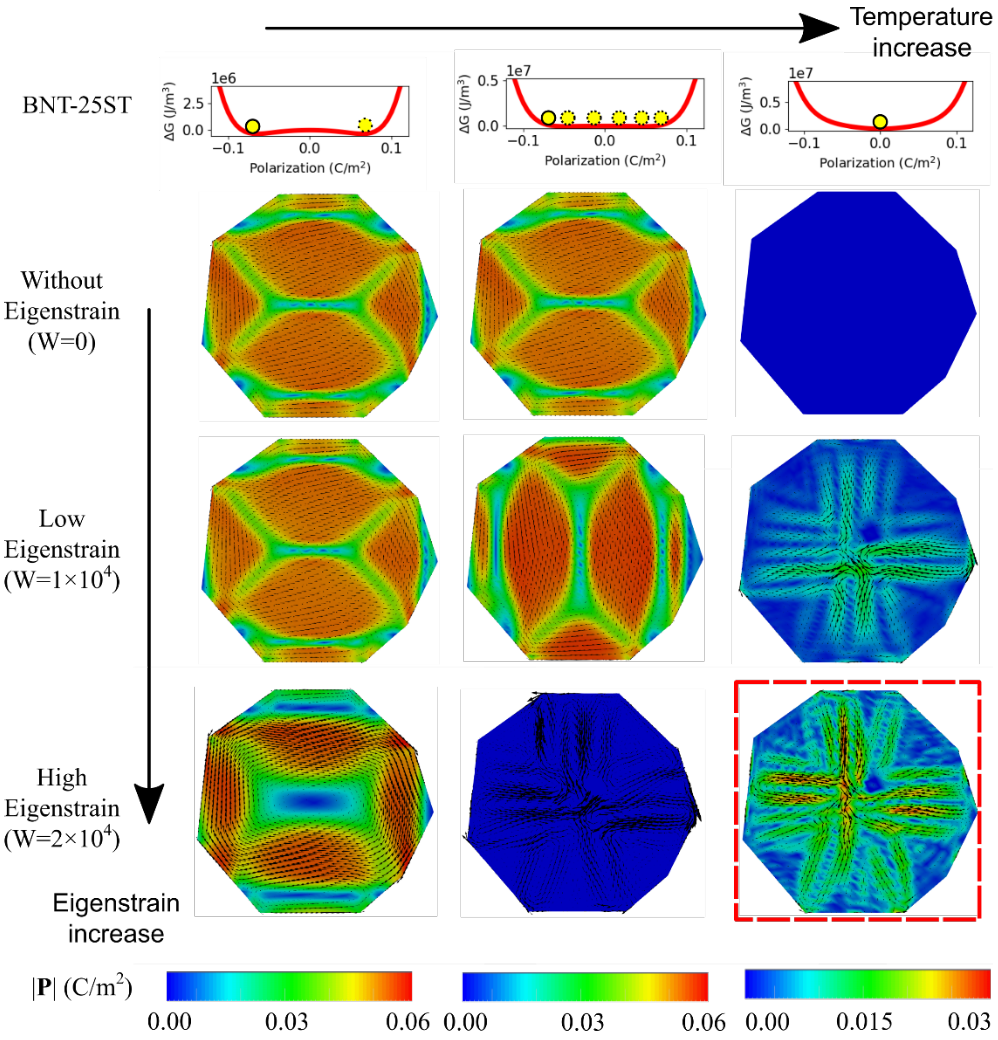

Supplementary Figure 8. Domain distribution of the nanoparticle at different temperature and eigenstrain values. The color shows the magnitude of the polarization and the arrows shows the direction of the polarization. The temperature increases from the left to the right column, while the eigenstrain increases from the top to the bottom row. The title row provides the corresponding Landau energy landscape ranges from a double well to a single well. The legends for the last column are different from the left two columns. The symbol |**P**| represents the magnitude of the polarization.

In order to show the influence of the temperature and the implemented eigenstrain on the polarization distribution, the following set of phase field simulations are given. The model is shown in the main article. Three characteristic temperatures (T = 100 °C, 200 °C and 800 °C, respectively) ranged from the tetragonal to cubic phase and three eigenstrain values are chosen for the simulation. For the case with lower temperature and without eigenstrain, the typical domain structure is formed with both 180° and 90° domain walls. Since charge free boundary condition is set at the edge of the nanoparticle, the polarization is oriented parallel to the boundary. At higher temperature (T =

500 °C) the nanoparticle is still in the tetragonal phase and the spontaneous polarization slightly decreases compared to the case with T = 25 °C, where the energy barrier H^{barrier} is lower. The domain structure remains almost the same. Above the Curie temperature (T_c ~ 540 °C), no polarization can be seen since the energy minimum is located at $|\mathbf{P}|$ = 0 C m^{-2}.

When a lower eigenstrain is implemented (second row), the domain structure is modified by the heterogeneous strain. More domains are formed in the second row compared to the first row. However, in the cubic phase, since the strain increases from the core to the shell, the orientation of the strain gradient is radial-shaped. As a result, the polarization points radially and a "seastar-like" polarization pattern is generated.

When a higher eigenstrain is implemented (third row), the influence of the eigenstrain on the domain patterns at T = 25 °C is weak, while it is larger at T = 500 °C. We have mentioned before that the Landau energy barrier is lower at T = 500 °C. This makes it easier to modify the domains by the external loading, e.g. mechanical strain. As a result, a "seastar-like" polar nanoregion is formed. Here, we give a rough estimation of the flexocoupling energy for a NBT-25ST nanoparticle. The flexocoupling tensor f is set in the magnitude of 1 V. With the eigenstrain factor $W = 2 \times 10^4$, the strain gradient is in the magnitude of 10^5. If the polarization is ~0.06 C m^{-2}, the flexocoupling energy $H^{\text{flexo}} = \sim 10^5$ J m^{-3} is in the same magnitude of H^{barrier}. At higher temperature, the polarization distribution is the same as in the low eigenstrain case, but the magnitude of the polarization is higher. The polarization distribution with different parameters (BTO and PTO) are calculated also. If readers are interested, please read the response to the reviewers.

Supplementary Note 7 Parameter determination for phase field simulation

Due to the lack of Landau parameter information for NBT-25ST system, we derive these values using the similar methods to Li *et al.*[5]

First, we assume the Landau energy under mechanical stress-free boundary condition take the form of a sixth-order polynomial,

$$H^{\mathrm{bulk}} = \alpha_1(P_1^2 + P_2^2) + \alpha_{11}(P_1^4 + P_2^4) + \alpha_{12}(P_1^2 P_2^2) + \alpha_{111}(P_1^6 + P_2^6) + \alpha_{112}(P_1^4 P_2^2 + P_2^4 P_1^2)$$

The coefficient α_1 is temperature dependent coefficient. For ferroelectrics, it is related to the permittivity by the Curie-Weiss law. In the paraelectric phase

$$2\alpha_1 = \frac{\partial^2 H^{\mathrm{bulk}}}{\partial P_1^2} = \frac{\partial E_1}{\partial P_1} = \frac{1}{\varepsilon} = \frac{T - T_0}{C} = 2\alpha_0(T - T_0)$$

where T_0 is the Curie-Weiss temperature and C is the Curie constant.

However, for relaxors, (e.g. NBT-25ST), the Curie-Weiss law is no longer valid. Instead, the permittivity follows the following law,

$$\frac{1}{\varepsilon} - \frac{1}{\varepsilon_{\mathrm{m}}} = \frac{(T - T_{\mathrm{m}})^{\gamma}}{C}$$

where γ is the diffusion factor which takes the value between 1 and 2.[6] We use the permittivity-temperature graph from Gomah-Pettry *et al.*[7] and fit it to the above modified Curie-Weiss law, the results are shown in Supplementary Figure 9. The diffusion factor $\gamma = 1.004$, which means that the dielectric behavior of this material is more like a ferroelectric. As a simplification, we assume $\gamma = 1$. The Landau coefficient:

$$\alpha_0 = 5.14 \times 10^5 (T - 216).$$

Then we attempt to determine the higher order Landau coefficients.

At Curie temperature, the Landau energy for paraelectric phase and ferroelectric phase are the same, i.e.

$$\alpha_0(T_{\mathrm{c}} - T_0)P_{\mathrm{sc}}^2 + \alpha_{11}P_{\mathrm{sc}}^4 + \alpha_{111}P_{\mathrm{sc}}^6 = 0$$

where P_{sc} is the spontaneous polarization at T_{c}.

On the other hand, at T_c

$$\left.\frac{\partial H^{\mathrm{bulk}}}{\partial P}\right|_{P=P_{\mathrm{sc}}} = 2\alpha_0(T_{\mathrm{c}} - T_0)P_{\mathrm{sc}} + 4\alpha_{11}P_{\mathrm{sc}}^3 + 6\alpha_{111}P_{\mathrm{sc}}^5 = 0$$

Combining the above two equations, one obtains,

$$T_{\mathrm{c}} - T_0 = \frac{-\alpha_{11}^2}{2\alpha_{111}\alpha_0}$$

$$P_{\mathrm{sc}}^2 = \frac{-\alpha_{11}}{2\alpha_{111}}$$

The value of P_{sc} is obtained from Krauss *et al.*[8] We regard the temperature with maximum permittivity T_m as T_c. Put all these values to the above equations, we get $\alpha_{11} = -1.25 \times 10^{10}\ \mathrm{C}^{-4}\ \mathrm{m}^6$ N and $\alpha_{111} = 3.91 \times 10^{12} \mathrm{C}^{-6}\ \mathrm{m}^{10}$ N.

The value of α_{12} and α_{112} depend on the transition temperature between Orthorhombic and Tetragonal. Unfortunately, for NBT-25ST, there is no such phase transition. According Watanabe et al., the Rhombohedral-Tetragonal phase transition temperature for NBT-25ST is 40 °C. If one assume 40 °C is the Orthorhombic-Tetragonal phase transition temperature, at such temperature

$$G(P_1 = 0; P_2 = P_\mathrm{t}) = G(P_1 = P_\mathrm{t}; P_2 = 0) = G(P_1 = P_2 = P_\mathrm{o})$$

and

$$\left.\frac{\partial H^{\mathrm{bulk}}}{\partial P_1}\right|_{P_1=P_2=P_0} = 2\alpha_0(T_\mathrm{c} - T_0)P_0 + 4\alpha_{11}P_0^3 + 6\alpha_{111}P_0^5 + 2\alpha_{12}P_0^3 + 6\alpha_{112}P_0^5 = 0$$

where P_t and P_o are the component of the spontaneous polarization in one direction for tetragonal and orthorhombic phase, respectively. Solving these two relationships simultaneously, one obtains the value of $\alpha_{12} = 3.52 \times 10^{10}$ $\mathrm{C}^{-4}\mathrm{m}^6$N and $\alpha_{112} = -1.25 \times 10^{12}$ $\mathrm{C}^{-6}\mathrm{m}^{10}$N. Fig. 2 shows the landau energy as a function of P_1 and P_2. The color indicates the free energy (H^{bulk}). Three temperatures 100 °C (at Tetragonal phase) 200 °C (near Curie temperature) and 800 °C (The operation temperature of the experiment) are chosen for the phase field simulation. The simulation results are shown in Fig. 3. One can find that the configuration of the polarization are almost the same to the previous calculation based on the modified BTO parameter, despite the magnitude of the polarization in tetragonal phase. Here we also listed the phase field simulation with the landau parameters for BTO and PTO for reference. The other parameters are identical. These results solidly prove that our simulation results and conclusion points are still valid despite the chosen of Landau parameter, especially in cubic phase.

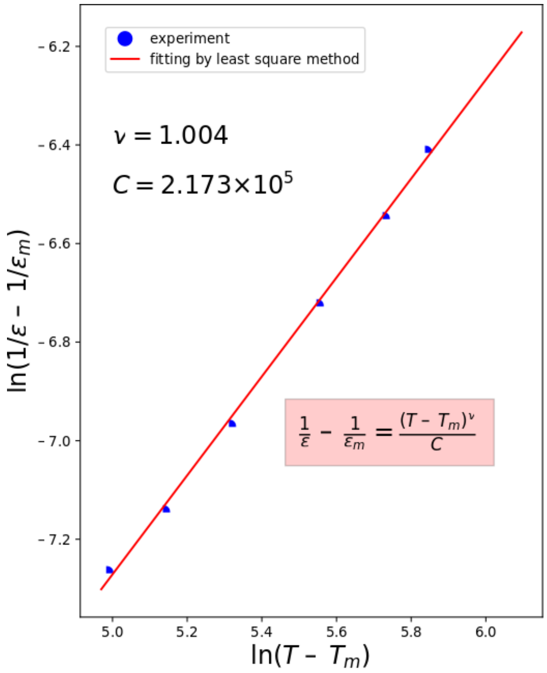

Supplementary Figure 9. Fitting curves for the relaxation parameter γ. The susceptibility as a function of temperature in logarithmic coordinates by the modified Curie-Weiss law for NBT-25ST. Blue dots: experimental data from Gomah-Pettry Jean-Richard *et al.*[7] Red line: fitting curve.

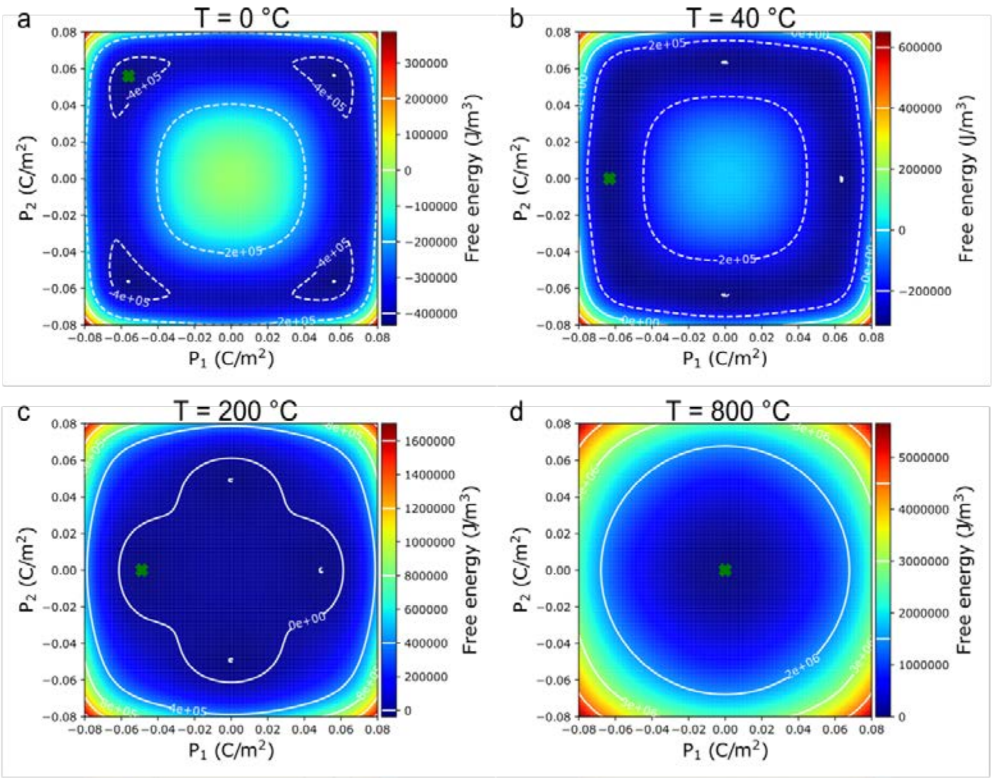

Supplementary Figure 10. The Landau energy as a function of the polarization by using the derived coefficients. a-d show the Landau energy at four different temperatures where the Rhombohedral-Tetragonal-Cubic phase transition can be captured.

The coefficients were modified according to the values given in Supplementary Table 1. The gradient energy coefficients were adopted from Hlinka *et al.*[9], where G_{11} = 51×10^{-11} J m^3 C^{-2}, G_{12} = -2×10^{-11} J m^3 C^{-2}, and G_{44} = 2×10^{-11} J m^3 C^{-2}. The dielectric tensor is based on NBT-06BT[10]. Here we assume an isotropic permittivity where k_{11} = ε and k_{12} = 0. For low temperature, where T/T_c = 0.37, the relative permittivity is ε = 1000. At T/T_c = 0.95, ε = 2000 and for higher temperature, T/T_c = 1.32, the Curie-Weiss law is used to get the permittivity with the value of 1328. The Young's modulus E = 120 GPa and Poisson ratio μ = 0.25.

**Coefficients**	**Value (*T* in K)**
α_1 **(10^5 C^{-2} m^2 N)**	5.14(*T*-489)
α_{11} **(10^{10} C^{-4} m^6 N)**	-1.25
α_{12} **(10^{10} C^{-4} m^6 N)**	3.52
α_{111} **(10^{12} C^{-6} m^{10} N)**	3.91
α_{112} **(10^{12} C^{-6} m^{10} N)**	-1.250

Supplementary Table 1. Landau coefficients used for the phase field simulation.

Supplementary Note 8 Mechanism of polarization generation in core-shell nanoparticles at elevated temperature starting from the chemical stoichiometry

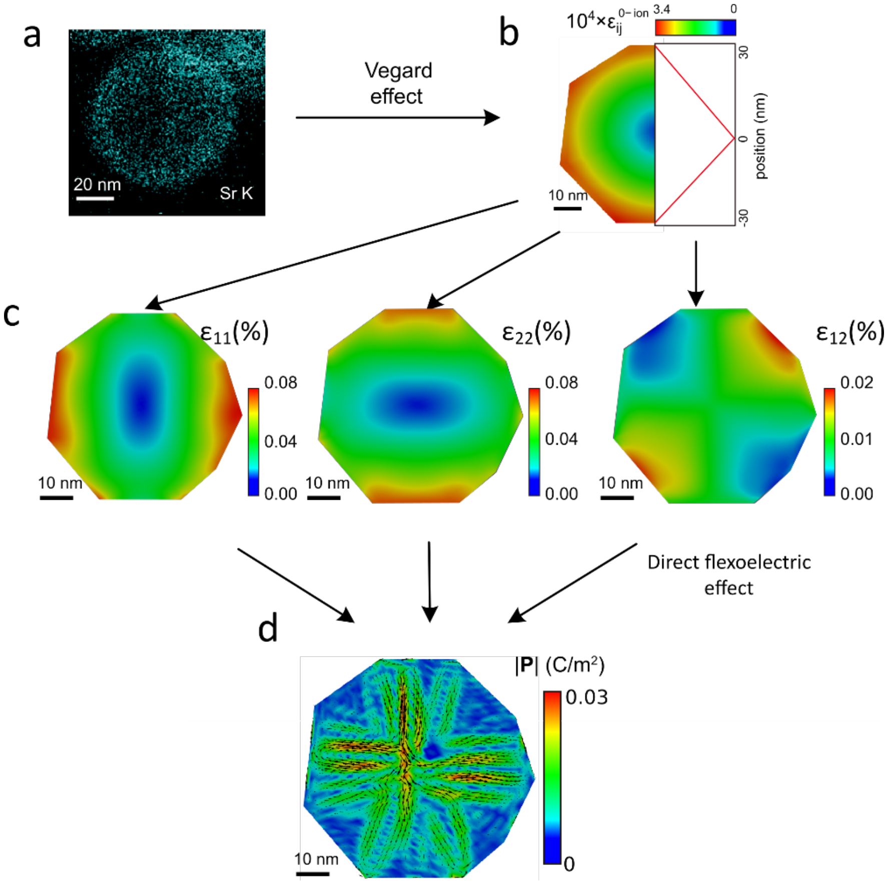

Supplementary Figure 11. Mechanism of polarization generation in core-shell nanoparticles at elevated temperature beginning from the local lattice distortion via the Vegard effect. The final polarization shown in **d** is the consequence of strain inhomogeneity (**c**) through the flexoelectric effect.

Supplementary Note 9 DLNRs with marked notations.

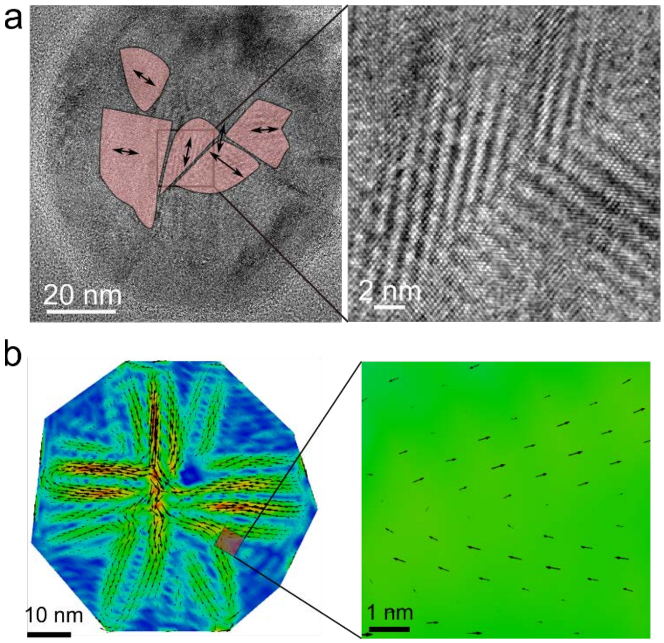

Supplementary Figure 12. The comparison between experiment and simulation. a, Bright-field transmission electron microscopy (TEM) image shows the domain-like nanoregions (marked red region) within the core-shell nanoparticle with enlargement figure shown at right-side. **b,** The simulation result with enlargement figure shown on the right.

Supplementary Note 10 Polarization from Chemical inhomogeneity.

In order to corroborate whether the local chemical fluctuations of the nanoparticle can lead to the formation of domain-like nanoregions at 800 °C, we performed a new simulation that considers the ferroelectric and flexoelectric contributions to the polarization in a nanoparticle with a random eigenstrain distribution. The random eigenstrain distribution is set to mimic the presence of local chemical fluctuations appearing in Supplementary Figure 13b. Except for the eigenstrain distribution, all the other parameters are exactly the same as in the simulation shown in Figure 3 in the manuscript. The random distributed eigenstrain is given by:

$$\varepsilon^0 = \begin{pmatrix} z & 0 \\ 0 & z \end{pmatrix}$$

where z is a random number in each simulated element, with the standard deviation of 2×10^{-5}. The real strain distribution is shown in Supplementary Figure 13a-c. The corresponding polarization distribution is shown in Supplementary Figure 13d. Note that the scale bar of Supplementary Figure 13d is the same as in Fig. 3e for comparison. The magnitude of the polarization is two orders of magnitude lower than the one calculated in Fig. 3c. Considering that all the other conditions are the same, it can be concluded that the radial strain distribution (ascribed to the Sr^{2+} chemical gradient) and its coupling with the flexoelectric effect are responsible for the domain-like nanoregions shown in Fig. 3e at 800 °C.

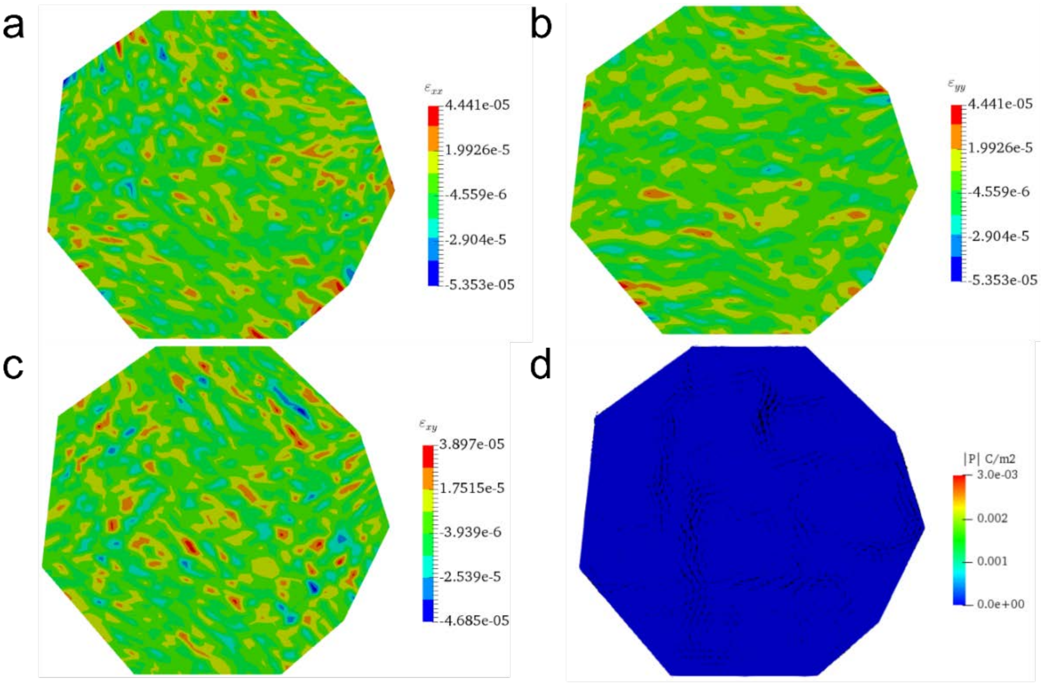

Supplementary Figure 13. Strain distribution with random distributed eigenstrain. **a-c** The strain component and **d** the corresponding polarization distribution based on the ferroelectric-flexoelectric model.

Supplementary References

1. Koruza, J. *et al.* Formation of the core–shell microstructure in lead-free $Bi_{1/2}Na_{1/2}TiO_3$-$SrTiO_3$ piezoceramics and its influence on the electromechanical properties. *J. Eur. Ceram. Soc.* **36,** 1009–1016 (2016).
2. Pérez-Garza, H. H. *et al.* MEMS-based sample carriers for simultaneous heating and biasing experiments: A platform for in situ TEM analysis. In Solid-State Sensors, Actuators and Microsystems (TRANSDUCERS), 19th *International Conference on. IEEE* 2155–2158 (2017).
3. Kilaas, R. Optimal and near-optimal filters in high-resolution electron microscopy. *J. Microscopy* **190**, 45-51 (1998).
4. Hÿtch, M. J. *et al.* Quantitative measurement of displacement and strain fields from HREM micrographs. *Ultramicroscopy* **74,** 131–146 (1998).
5. Li, Y. L., Cross. *et al.* A phenomenological thermodynamic potential for $BaTiO_3$ single crystals. *J. Appl. Phys.* **98,** 064101 (2005).
6. Viehland, D., & Li, J. F. Compositional instability and the resultant charge variations in mixed B ‐ site cation relaxer ferroelectrics. *J. Appl. Phys.*, **74**, 4121-4124 (1993).
7. Gomah-Pettry, J. R, *et al.* Sodium-bismuth titanate based lead-free ferroelectric materials. *J. Eur. Ceram. Soc.*, **24**, 1165-1169 (2004).
8. Krauss, W. *et al.* Piezoelectric properties and phase transition temperatures of the solid solution of $(1-x)(Bi_{0.5}Na_{0.5})TiO_3$–$xSrTiO_3$. *J. Eur. Ceram. Soc*, **30**, 1827-1832 (2010).
9. Hlinka, J. & Márton, P. Phenomenological model of a 90° domain wall in $BaTiO_3$-type ferroelectrics. *Phys. Rev. B* **74,** 104104 (2006).
10. Rödel, J. *et al.* Perspective on the Development of Lead-free Piezoceramics. *J. Am. Ceram. Soc.* **92,** 1153–1177 (2009).
