# Supplementary material for: Enabling nanoscale flexoelectricity at extreme temperature by tuning cation diffusion
Source: Nat Commun. 2018 Oct 25;9:4445. doi: 10.1038/s41467-018-06959-8 (PMC6202390; doi:10.1038/s41467-018-06959-8)
Supplement: Supplementary file 3 — Description of Additional Supplementary Files [file 41467_2018_6959_MOESM3_ESM.pdf]

**Description of Additional Supplementary Files**

**File Name:** Supplementary Movie 1

**Description:** Core-shell nanoparticle dynamics under electric-field
